# Nelfinavir Inhibition of Kaposi’s sarcoma-associated herpesvirus protein expression and capsid assembly

**DOI:** 10.21203/rs.3.rs-3552962/v1

**Published:** 2023-11-08

**Authors:** Maggie Li, Barbara Smith, Lee Jaeyeun, Jennifer Petr, Robyn Wiseman, Nicole Anders, Michelle Rudek, Richard Ambinder, Prashant Desai

**Affiliations:** Johns Hopkins University; Johns Hopkins University; Johns Hopkins University; Johns Hopkins University; Johns Hopkins University; Johns Hopkins University; Johns Hopkins University; Johns Hopkins University; Johns Hopkins University

**Keywords:** Kaposi’s sarcoma associated herpesvirus, Nelfinavir, virus inhibition, integrated stress response, virus assembly

## Abstract

**Background:**

Antiviral therapies that target herpesviruses are clinically important. Nelfinavir is a protease inhibitor that targets the human immunodeficiency virus (HIV) infections aspartyl protease. Previous studies demonstrated that this drug could also inhibit Kaposi’s sarcoma-associated herpesvirus (KSHV) production. Our laboratory demonstrated nelfinavir can effectively inhibit herpes simplex virus type 1 (HSV-1) replication. For HSV-1 we were able to determine that virus capsids were assembled and exited the nucleus but did not mature in the cytoplasm indicating the drug inhibited secondary envelopment of virions.

**Methods:**

For KSHV, we recently derived a tractable cell culture system that allowed us to analyze the virus replication cycle in detail. We used this system to further define the stage at which nelfinavir inhibits KSHV replication.

**Results:**

We discovered that nelfinavir inhibits KSHV extracellular virus production. This was seen when the drug was incubated with the cells for 3 days and when we pulsed the cells with the drug for 1–5 minutes. When KSHV infected cells exposed to the drug were examined using ultrastructural methods there was an absence of mature capsids in the nucleus indicating a defect in capsid assembly. Because nelfinavir influences the integrated stress response (ISR), we examined the expression of viral proteins in the presence of the drug. We observed that the expression of many were significantly changed in the presence of drug. The accumulation of the capsid triplex protein ORF26 was markedly reduced. This is an essential protein required for herpesvirus capsid assembly.

**Conclusions:**

Our studies confirm that nelfinavir inhibits KSHV virion production by disrupting virus assembly and maturation. Of interest is that inhibition requires only a short exposure to drug. The source of infectious virus in saliva has not been defined in detail but may well be lymphocytes or other cells in the oral mucosa. Thus, it might be that a “swish and spit” exposure rather than systemic administration would prevent virion production.

## Introduction

Kaposi sarcoma is a tumor associated with Kaposi’s sarcoma-associated herpesvirus (KSHV also known as HHV-8). Transmission is believed to be salivary in most instances [1–6]. Agents that inhibit KSHV lytic replication have not been shown to be effective in the treatment of tumors [7]. However, agents that inhibit production of lytic virus might block salivary transmission [8]. Oral valganciclovir reduces KSHV shedding. Although well tolerated in the short term [8], valganciclovir is often associated with myelosuppression when used for long term treatment [9].

A topical therapy might inhibit lytic replication and viral transmission without associated systemic side effects. There is precedence for exploring topical agents to interfere with viral transmission. The Hendrix Lab has explored the use of rectal enemas with a variety of antiviral agents to block HIV transmission [10, 11].

Nelfinavir was developed as a protease inhibitor targeting the human immunodeficiency virus (HIV) aspartyl protease [12–14]. However, nelfinavir often leads to diarrhea [15]. With the advent of HIV protease inhibitors that are better tolerated, nelfinavir is rarely used in the treatment of HIV. A series of studies showed a variety of poorly understood off-target effects of nelfinavir [16]. Among them was a report showing antiviral activity against human herpesviruses [17]. This report led us to investigate the effects of nelfinavir on HSV-1. We found that nelfinavir does not affect the activity of HSV-1 maturation protease; however, it alters glycoprotein maturation [18, 19]. We presented ultrastructural evidence that HSV type 1 infected cells treated with nelfinavir did not release virions into the intercellular space, but rather accumulated unenveloped virion particles in the cytoplasm [19]. The mechanism was unclear but other studies showed modulation of the unfolded protein response (UPR), cell cycle, apoptosis, autophagy, the proteasome pathway, oxidative stress and the integrated stress response (ISR) [20–23]. Phosphorylation of the translation initiation factor eIF2α to decrease overall translation initiation and increase production of stress factors including the transcription factor ATF4 is one of the hallmarks of ISR [24].

Gantt *et al*. reported that nelfinavir inhibited KSHV release by cells in tissue culture with an EC_50_ of 7.4 μM (3.5 times more potent than ganciclovir) [25]. Because nelfinavir has shown promising results for KSHV inhibition, we theorized that it could be used to inhibit KSHV viral shedding. Since the site of shedding, the oral mucosa, is readily accessible, we hypothesize that a local antiviral formulation might inhibit shedding with minimal systemic exposure. Local antiviral drug delivery might involve a mouthwash or “swish and spit” therapy, that would reduce KSHV shedding in the saliva, and salivary transmission to uninfected partners. We sought to investigate further how nelfinavir may inhibit KSHV virus replication using a more tractable cell culture system and a recombinant KSHV virus developed by Jeffery Vieira [26]. In the investigations described in this report, we examine the activity of nelfinavir with regard to KSHV and explore the effects of brief drug exposures on virus excretion such as might be achieved with a microbicidal mouthwash.

## Material and Methods

### Cells and Viruses

All cell lines were grown in minimal essential medium (alpha medium – Gibco Invitrogen) supplemented with 10% fetal bovine serum (FBS – Gibco Invitrogen) and passaged as described previously [27]. The Vero cell line carrying the recombinant rKSHV.219 virus and the recombinant baculovirus BacK50 were obtained from Jeffery Vieira [26]. These cells were used to sub-clone a Vero line that displayed almost 100% GFP positivity in the presence of puromycin (5 μg/ml). The cell line iSLK developed by the Ganem Lab [28] was obtained from Jae Jung. 5r219 cells were maintained in 10 μg/ml puromycin continuously.

### Generation of an iSLK cell line harboring rKSHV.219

We created a KSHV positive iSLK cell line for these studies. To do this we derived virus from the Vero cell line harboring the KSHV recombinant virus rKSHV.219 [26] to infect the RTA-inducible iSLK cell line [28]. To get KSHV virus from the Vero cell line, we infected 1 × 10^6^ cells with BacK50 (baculovirus expressing ORF50-RTA)[26] for 3 hours. This virus was removed and sodium butyrate added at a concentration of 0.5 mM in the growth medium. Four days following infection, the virus containing supernatant was used to infect 1 × 10^6^ iSLK cells. The cells were monitored for the expression of GFP and four days following infection when more GFP positive cells were evident, the culture was trypsinized, diluted and plated in media containing, initially high concentration (20 μg/ml) and then lower concentration (10 μg/ml) of puromycin. Individual GFP positive: puromycin resistant colonies were harvested using cloning cylinders and established lines were derived. One such cell line designated 5r219 was used for all subsequent studies.

### KSHV genome qPCR

Standards were created using KSHV DNA from the JSC-1 cell line [29] and all manipulations were done on ice. The PCR Master Mix was made using qPCR Taqman Universal Master Mix II (Thermo Fisher), 10 μM working stocks of the ORF73 Forward (5’-CCAGGAAGTCCCACAGTGTTC-3’) and Reverse Primers (5’-GCCACCGGTAAAGTAGGACTAGAC-3’), 1 μM working stock of ORF73 Fluorescent Probe (5’/56-FAM/CATCCGGGCTGCCAGCATTTG/36-TAMSp/3’), and dH_2_O and vortexed for 10 seconds. The wells of a 96-well PCR plate were loaded with 40 μL of the PCR Master Mix and 10 μL of the standards, RNase/DNase-free H_2_O as a negative control, or virus supernatant samples. The plate was sealed with optical sealing tape and placed into the Bio-Rad CFX 96 qPCR Detection System. The thermal cycling parameters from the TaqMan Universal Master Mix II Protocol (Thermo Fisher) were used.

### KSHV GFP Titration

A GFP titration assay was generated by concentrating the virus present in the supernatant of a well (12 well size 1 × 10^6^ cells) by centrifuging in a microfuge at 14000*g*for 60 min at +4°C. The supernatant was discarded and the virus resuspended in PBS (100 μl) and kept at + 4°C overnight. The following day the virus suspension was vortexed for 20 seconds and the whole 100 μI volume added to Vero/293T cell monolayers in 12 well trays. The virus was absorbed to the cells for 2 h with intermittent shaking. Fresh media was added to the cells after the virus inoculum was removed. Cells were imaged in a Zoe (Bio-Rad) fluorescence microscope after 3 days.

### Cell viability Assays

5r219 cells (1 × 10^6^) were incubated with the drug for the duration and concentration required. After treatment, the cells were washed with PBS, trypsinized and resuspended in a final volume of 100 μI and transferred to 96 well tray. An equal volume of CellTiter-Glo reagent (Promega) was added to the cells, mixed on a shaker for 2 min and incubated at room temperature for 10 min before the luminescence signal was read in a GloMax plate reader (Promega).

### Intracellular drug concentration

Nelfinavir was quantified in iSLK cells immediately after treatment and at 24, 48 and 72 hours. Methanol (200 μL) was added to the pelleted iSLK cells before extraction. The standard curve and quality control samples were prepared in methanol as a surrogate matrix for all matrices. Nelfinavir was extracted from 20 μL of sample with 80 μL of acetonitrile containing 125 ng/mL of the internal standard, ritonavir-d6. After centrifugation, the supernatant was then transferred into autosampler vials for LCMS/MS analysis. Separation was achieved with an UPLC BEH C18 (2.1×50mm, 1.7μm) column with a gradient elution using 0.1% formic acid in water (v/v; mobile phase A) and 0.1% formic acid in acetonitrile (v/v; mobile phase B). The flow rate was kept constant at 0.2 mL/min and the gradient started with 40% mobile phase B. Mobile phase B was held at 40% for 0.5 minute before increasing to 100% mobile phase B over the span of 1.0 minute. Next, mobile phase B was held at 100% for 1.0 minute and then returned back down to 40% over 0.1 minute. Finally, mobile phase B was allowed to equilibrate for 0.4 minute for a total run time of 3.0 minutes. The column effluent was monitored using an AB Sciex triple quadrupole^™^ 4500 mass-spectrometric detector (Sciex, Foster City, CA, USA) using electrospray ionization operating in positive mode. The spectrometer was programmed to monitor the following MRM transitions: 569.1 → 330.0 for nelfinavir and 727.3 → 302.2 for ritonavir-d6. Calibration curves for nelfinavir were computed using the area ratio peak of the analysis to the internal standard by using a quadratic equation with a 1/x^2^ weighting function over the calibration ranges of 1.5 to 736.3 nM with dilutions up to 1:10 (v:v). Results were expressed in nmol/10^6^ cells.

### RT qPCR

RNA was extracted from cell pellets using the Qiagen RNeasy Kit according to manufacturer instructions. The resulting RNA samples were quantified using a NanoDrop 2000 (Thermo Fisher). cDNA was synthesized using the Bio-Rad iScript cDNA Synthesis Kit according to manufacturer instructions. Three PCR Master Mixes were created to detect DNA sequences associated with the GAPDH, CHOP and Trib3 genes. Each of the master mixes contained the Bio-Rad SsoFast EvaGreen supermix, RNase/DNase-free water, and forward and reverse primers [30, 31] associated with each gene. The wells of a 96-well PCR plate were loaded with 18 μL of one of the PCR Master Mixes and 2 μL of cDNA sample. Each sample had 2 or 3 technical replicates for each of the 3 genes being detected. The plate was sealed with optical sealing tape and placed into the Bio-Rad CFX 96 qPCR Detection System. The thermal cycling parameters from the SsoFast EvaGreen protocol were used.

### Immunoblots

5r219 cells (1 × 10^6^) were harvested 72 h post-induction. Cell pellets were lysed in 2X Laemmli buffer and 10% of this sample was resolved using NuPAGE 4–12% Bis-Tris gels (Thermo Fisher) and transferred to nitrocellulose membranes using the iBlot2 system (Thermo Fisher), as described by Luitweiler *et al*. [32]. Rabbit antibodies to KSHV proteins were used at a dilution of 1:500. Mouse antibody to ORF26 was purchased from Novus. Rabbit polyclonal to GAPDH (Invitrogen) was used at 1:2500 dilution. Blots were processed using the Clarity chemiluminescence kit (Bio-Rad) according the manufacturer’s protocol and imaged using the iBright Imager (Invitrogen).

### Transmission Electron Microscopy (TEM)

5r219 cells (1 X 10^6^) cells were induced for lytic activation using 1 μg/ml doxycycline for 60 min. The cells were then incubated with nelfinavir (15 and 20 μM) for 72 h. No drug controls were also included. Samples were fixed in 2.5% glutaraldehyde, 3mM MgCl_2_, in 0.1 M sodium cacodylate buffer, pH 7.2 for overnight at 4°C. After buffer rinse, samples were postfixed in 1% osmium tetroxide in 0.1 M sodium cacodylate buffer (1 h) on ice in the dark. Following a DH_2_O rinse and en bloc staining in 0.75% uranyl acetate for three hours, samples were dehydrated in a graded series of ethanol and embedded in Eponate resin overnight at 60°C. Thin sections, 60 to 90 nm, were cut with a diamond knife on a Leica UltracutE ultramicrotome and picked up with 2×1 mm formvar coated copper slot grids. Grids were stained with 2% uranyl acetate (aq.) and 0.4% lead citrate before imaging on a Hitachi 7600 TEM at 80 kV equipped with an AMT XR80 CCD.

## Results

### Generation of 5r219 cell line

**Figure 1:**
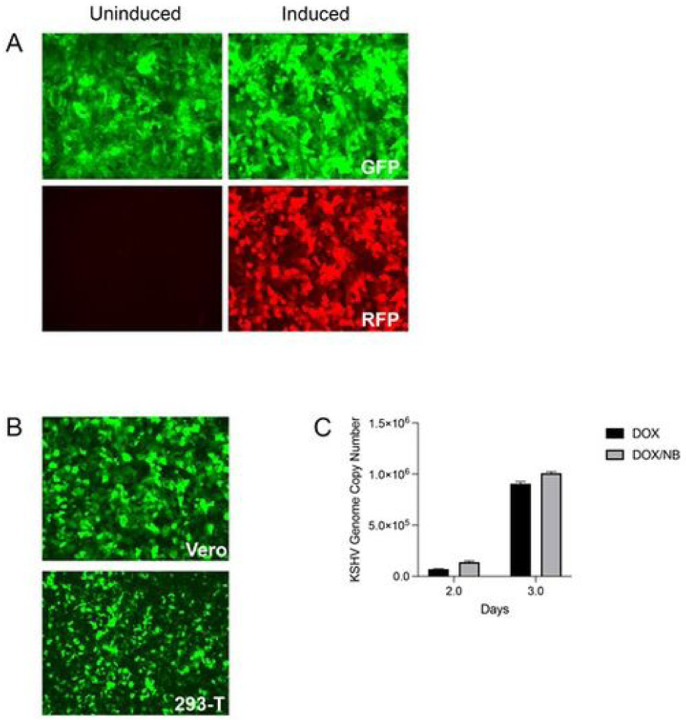
Establishment of a tractable virus producer cell line for KSHV r219. The 5r219 cell line was generated following infection of iSLK cells with KSHV.r219 virus derived from Vero cells. Clonal isolates that displayed the highest level of lytic induction were analyzed further. The cells were first treated with doxycycline (1 mg/ml) and fluorescence visualized by light microscopy. Red fluorescence was only observed after lytic induction (A). The supernatants from these cultures were harvested at 72 hours, concentrated by centrifugation and the virus was used to infected monolayers of Vero or HEK-293T cells. Numerous GFP positive cells were observed 48h post-infection indicative of KSHV virus infection (B). In order to quantitate virus yields and the optimal time of virus production, 5r219 cells were induced with doxycycline or doxycycline plus sodium butyrate (1 mM) and virus supernatants harvested, 2 and 3 days post-induction, and used in qPCR assays to determine KSHV genome copies (C).

We wished to examine the antiviral activity of nelfinavir in greater detail using a more tractable cell culture system for KSHV. To this end we re-created an inducible SLK cell line harboring KSHV, similar to iSLK.219 cl.10 that was originally described by Myoung and Ganem [28]. The KSHV recombinant rKSHV.219 was originally isolated by Jeff Vieira [26]. This virus expresses GFP constitutively (EF1 a promoter) and upon lytic induction expresses RFP driven by the lytic KSHV PAN promoter. It also carries a puromycin resistance gene. Previously, we had obtained the Vero rKSHV-219 cell line from Jeff Vieira. This line requires infection with a recombinant baculovirus expressing ORF50 (RTA) and sodium butyrate treatment for lytic induction [26]. This was not conducive for our experiments. Therefore, we derived rKSHV.219 virus from the Vero cell line (after lytic induction) and infected iSLK cells. These cells express RTA under control of a doxycycline (DOX)-responsive promoter, the tetracycline-responsive element (TRE), for more efficient lytic induction [28]. Following infection of iSLK cells we derived cell lines that were GFP positive and puromycin resistant. Individual clones were selected for their lytic inducibility (RFP fluorescence) following the addition of doxycycline. One such clonal cell line designated 5r219 was used for all our experiments (Fig. 1A). Further experiments were used to show the production of KSHV virus following induction with DOX alone or with DOX and sodium butyrate. We quantitated virus production using a GFP titer assay on Vero and 293T monolayers (Fig. 1B) as well as a qPCR assay to quantitate viral genomes produced (Fig. 1C). Our findings showed that we could achieve significant virus yield at 3 days post-induction and we eliminated sodium butyrate from the induction because of the toxicity of this compound.

### Inhibitory activity of nelfinavir on KSHV replication

**Figure 2:**
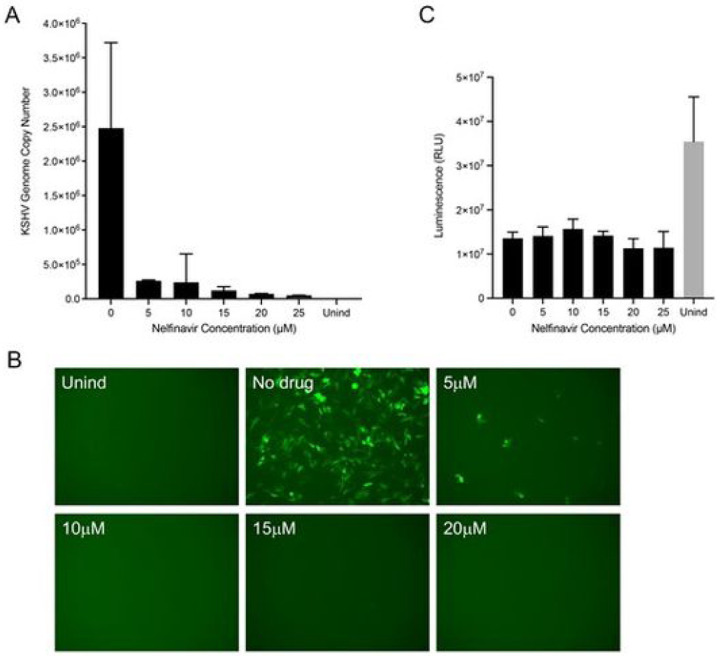
Nelfinavir inhibits KSHV virus production. The effect of nelfinavir on KSHV virus production was examined using a standard dose-response assay. 5r219 cells were first incubated with media containing 1 mg/ml doxycycline for 1 h. After that replicate cultures were incubated with varying concentrations of nelfinavir in the continued presence of doxycycline for 72 h. The culture supernatants were harvested, clarified and used in qPCR assays (A) as well as concentrated and pelleted virus used to infect Vero cell monolayers (B). Similar 5r219 cell cultures were examined for cell viability using the CellTiter-Glo assay (C).

We first tested the effect of nelfinavir at different concentrations of drug. Using qPCR, we observed inhibition of genome copy at concentrations higher than 10 μM (Fig. 2A). There was a 90% reduction in virus copies at 15 μM. We also examined the inhibition of virus production using the GFP titer assay (Fig. 2B). In the absence of the drug, numerous GFP positive cells were evident, indicative of robust virus production. Addition of nelfinavir significantly decreased the number of GFP positive cells. We also tested the toxicity of the drug on replicate cultures, and again the data showed there was minimal toxicity even at the high concentrations of the drug (Fig. 2C). This was similar to what we observed with Vero cells [19]. Gantt *et al*. reported 80% inhibition of KSHV virus production using 10 μm nelfinavir [17].

### High-dose short duration exposure of KSHV infected cells to nelfinavir

**Figure 3:**
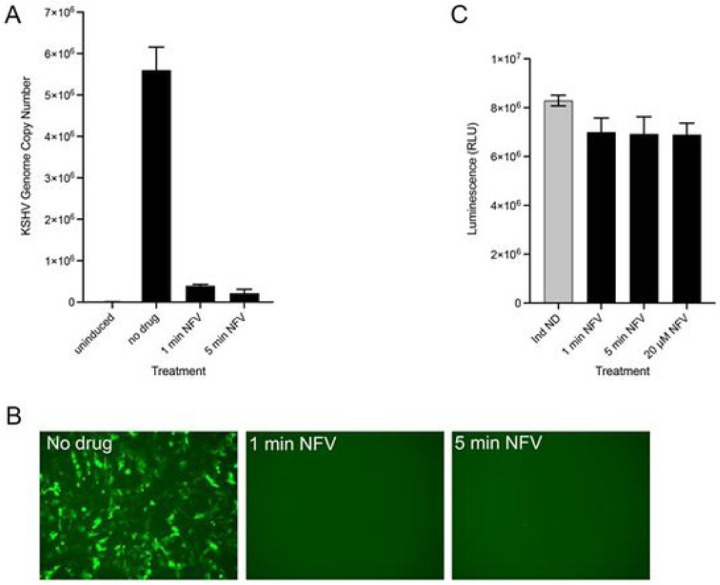
High-dose short-duration exposure of nelfinavir and KSHV inhibition. 5r219 cells were lytically induced with doxycycline. After 1 hour induction, the cells were treated with 1 mM nelfinavir mesylate for 1 minute or 5 minutes. The drug was removed and the cells were incubated in medium containing only doxycycline. The cell culture supernatants were harvested at 72 hours and viral genomes quantified using qPCR assays (A). Virus was also concentrated and the pelleted virus used to infect Vero cells. Fluorescence was examined after 48 h (B). Cell viability was examined for similarly treated cultures and compared to cells incubated with 20 mM nelfinavir continuously for 72 h (C).

In the above experiment the drug was incubated with the cells for 3 days at low micromolar concentrations. We then began to look at short duration exposure of high doses of nelfinavir and ganciclovir and its effect on KSHV production and cell toxicity. We initially started with 30 min exposure using 1 mM nelfinavir and 10 mM ganciclovir. The cells were exposed both after 1 h post-lytic induction (+ DOX) and after 20 h post-lytic induction. We observed significant decrease in virus yield as judged by the GFP titer assay when the drug was incubated for only 30 min (data not shown). The inhibitory effect was more pronounced when the cells were treated after 1 h post-lytic induction (data not shown). This led us to try even shorter durations of drug exposure. We tested various short pulses of drug treatment. We observed that virus production was inhibited by very short pulses of nelfinavir, but for ganciclovir we had to treat the cells for longer times to achieve similar inhibition (data not shown). We settled on exposing the cells for 1 min and 5 min of 1 mM nelfinavir only, as it was more potent. The intracellular drug concentrations remained consistent over 72 hours and were 4.0 ±1.3 nmol/10^6^ cells after 1 min of treatment and 6.7 ± 3.8 nmol/10^6^ cells after 5 min of treatment. This treatment was as effective at inhibiting KSHV virus production as the continuous exposure of drug (Fig. 3A–B). Yields of virus were reduced by greater than 90%. Even at the high doses of nelfinavir used, the cells displayed minimal cell toxicity as judged by viability assays (Fig. 3C).

### Nelfinavir induces the UPR in 5r219 cells

**Figure 4:**
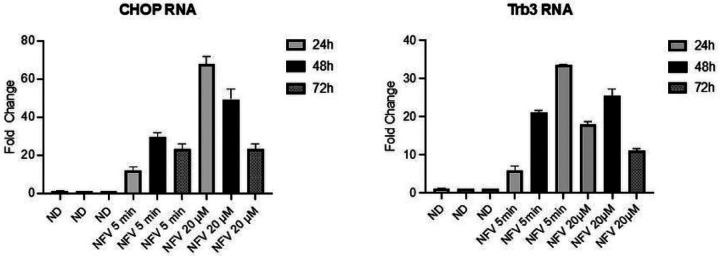
Nelfinavir induces gene expression of CHOP and Trb3. 5r219 cell cultures were induced with doxycycline and then exposed to 1 mM of nelfinavir for 5 mins or 20 mM nelfinavir for 72 h. At 24, 48 and 72 h after induction, RNAs were extracted from replicate cultures and were analyzed using RT qPCR methods

Because several studies have shown that nelfinavir is a potent inducer of the UPR [21], we wished to confirm that the same was true in the cell culture system that we have used in this study and with the short duration drug treatment. We performed an RT qPCR assay to investigate this (Fig. 4). Using RT qPCR assays we observed both CHOP and Trib3 RNA levels increase significantly following nelfinavir treatment for 5 min (1 mM) or nelfinavir treatment for 72 h (20 μM).

### Ultrastructural analysis of nelfinavir treated cells

**Figure 5:**
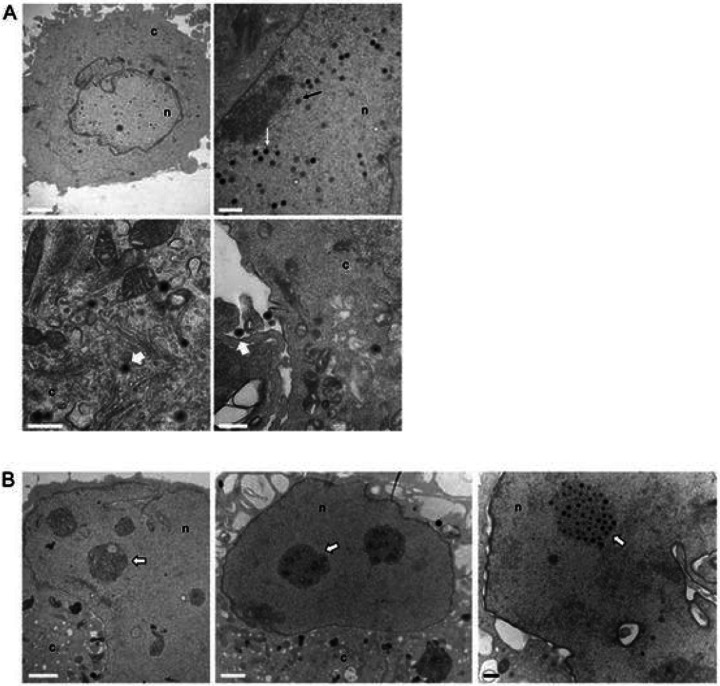
Ultrastructural analyses of nelfinavir treated 5r219 cells. 5r219 cells were induced with doxycycline and then exposed to 20 mM nelfinavir for 72 h. Cells were then processed for thin section and imaged by TEM. In the no drug controls (A), capsid structures were evident in the nucleus. Some contained an electron dense DNA core (white arrow) whereas others had an internal scaffold core (black arrow). In the cytoplasm and at the cell membrane, enveloped virions were evident (white arrowhead). In the nelfinavir treated cells (B), mature capsids were not observed however, within the nucleus, large dense aggregations were evident (white arrowheads). Scale bar = 1000 micron. c - cytoplasm, n - nucleus.

We sought to determine where the block in KSHV virus production occurs in nelfinavir-treated cells. We treated 5r219 cells with nelfinavir and then processed and evaluated the cells by transmission electron microscopy (TEM). Enveloped virions and capsids were evident in the no drug (control) cell cultures (Fig. 5A). However, in nelfinavir-treated cells we did not see normal capsid assembly (Fig. 5B). Normal nuclear capsids were evident in the no drug cultures, but they were not evident in the nucleus of nelfinavir-treated cells. Instead, we observed nuclear aggregations, likely virus assembly compartments [33]. It is here that capsids would mature and become packaged with DNA. These assembly compartments do not appear to facilitate the maturation of nuclear capsids.

### KSHV virus protein expression in nelfinavir treated cells

**Figure 6:**
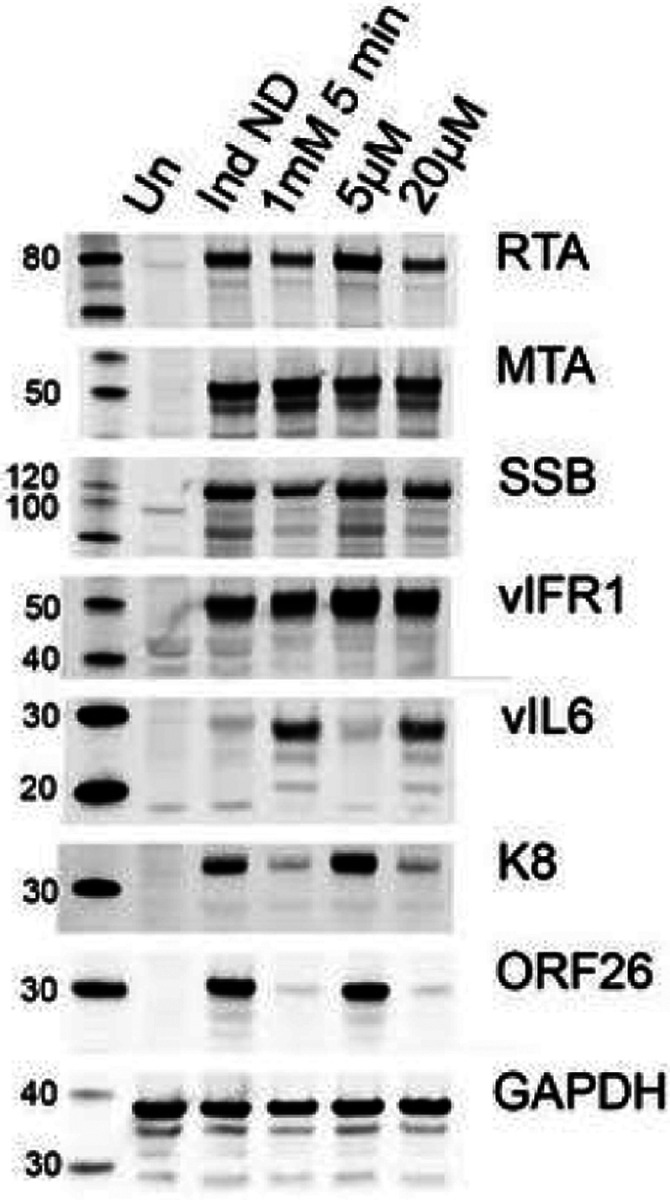
Effects of nelfinavir on KSHV protein expression. 5r219 cultures were induced with doxycycline and exposed to nelfinavir (1 mM for 5 minutes or 5 and 20 mM of drug for 72 h). The cells were harvested at 72 h post-induction and the proteins were analyzed by western blot methods. Molecular weight standards are shown in the left lane. Un: uninduced, Ind ND: Induced with doxycycline no drug control.

Nelfinavir is a potent inducer of the ISR [20]. It has been shown that nelfinavir can decrease overall translation rates and facilitate transcriptional activity characteristic of the ISR [20]. We have also shown this recently with the antimicrobial drug, clofoctol and arsenic, in EBV positive cell lines [30, 31]. This may be one of the mechanisms by which nelfinavir inhibits virus capsid maturation: by altering the expression of an essential structural protein. We thus examined protein expression levels in 5r219 cells exposed to nelfinavir (both short pulse and continuous) using immunoblot methods. We chose several KSHV antigens to examine, some expressed early and others expressed late in the replication cycle. The data are shown for uninduced cells as well as induced cells (+ DOX), cells treated with a 5 min pulse of 1 mM (inhibitory) nelfinavir and cells treated continuously with 5 μM (not inhibitory) and 20 μM (inhibitory) nelfinavir (Fig. 6). For proteins such as MTA (ORF57) and vIRF1 there was minimal change in protein accumulation. For RTA and SSB (ORF6) there was a noticeable decrease in protein accumulation in the presence of inhibitory concentrations of nelfinavir. Interestingly, for vIL6 there was an increase in protein detected in the presence of nelfinavir concentrations that inhibit virus production. The most significant effect is on K8 and the capsid triplex protein, ORF26. The levels of these proteins detected was significantly diminished in the presence of inhibitory concentrations of nelfinavir.

## Discussion

In this study we used a more tractable culture system for KSHV to investigate how this drug prevents virion formation. Our data show that for KSHV, inhibition occurs at a stage earlier than capsid assembly. In the nuclei of cells treated with this drug, we observed large nuclear protein aggregates which are akin to virus assembly compartments seen in herpesvirus-infected cells [33]. These electron-dense bodies represent sites where virus proteins accumulate and begin the assembly of the capsid and subsequent DNA packaging of the assembled capsids. We did not observe any mature capsids in the nucleus. In cells not treated with nelfinavir we observed capsids with internal scaffold core and DNA cores. Thus, nelfinavir prevents capsid formation in this KSHV cell culture system. This was different to what was observed in HSV-1 infected cells: nelfinavir did not affect capsid assembly and DNA packaging, nor did it affect nuclear egress of mature capsids, but rather an essential step in the cytoplasm during secondary envelopment [19].

These different observations of how nelfinavir inhibits herpesvirus production could be related to the differences in how the two viruses replicate and the optimal cell culture system that each virus is grown in. We believe, for KSHV it is the effect of the drug on the ISR [24, 34] which then manifests as a block in virus capsid assembly. This was evident in the viral protein analysis which showed that the accumulation of some KSHV proteins was inhibited by nelfinavir. The accumulation of RTA, the potent transactivator of KSHV, was reduced by the drug but not abolished, hence, its ability to turn on lytic genes was still active as demonstrated by the expression of lytic proteins. However, some viral proteins showed significantly inhibited accumulation. This was observed for ORF26 which is an essential capsid protein. ORF26 is part of the triplex complex of the capsid shell. There are two copies of ORF26 in complex with the other triplex protein, ORF62, and this trimer has been shown to be essential for herpesvirus capsid assembly. If either is absent, capsids fail to assemble [35–41]. Hence, it seems likely that with the significant reduction in ORF26 protein accumulation, capsid assembly was abolished. This phenotype, perturbation of the ISR, was also observed when clofoctol or arsenic, was used in EBV-infected cells [30, 31]. The expression of late structural proteins was also substantially decreased.

Previous studies in the lab have shown nelfinavir can induce the lytic replication cycle of KSHV in PEL cell lines and EBV in Burkitt lymphoma (BL) (data not shown). In those experiments, addition of 20 μM nelfinavir resulted in elevated levels of ATF4, XBP-1 and CHOP-10 indicative of ER stress. Nelfinavir was also shown to induce the expression of the major transactivators of EBV (ZTA) and KSHV (RTA). This observation is because of the effect of the drug on ER stress and the UPR, similar to that observed with tunicamycin and thapsigargin [23, 42]. We similarly observed in the 5r219 cells that incubation with nelfinavir induced lytic gene expression as judged by expression of RFP from the Pan promoter. The mechanism of how the drug modulates the latency program to initiate lytic is unclear. Nelfinavir promotes phosphorylation of eIF2a which leads to increased expression of ATF4 and increased expression of known downstream targets genes [20]. The ISR leads to general decrease in mRNA translation and thus global reduction in protein translation [24, 34]. These events likely disrupt virus assembly and maturation because of their impact on the synthesis of essential viral proteins.

In conclusion, our studies confirm the initial observations of Gantt *et al*. [17] that nelfinavir inhibits KSHV virion production by disrupting virus assembly and maturation. A particularly interesting aspect of this inhibition is that it requires only a short exposure to drug. The source of infectious virus in saliva has not been defined in detail but may well be lymphocytes or other cells in the oral mucosa. Thus, it might be that a “swish and spit” exposure rather than systemic administration would prevent virion production. Much work remains to be done to better understand the mechanism of action of the antiviral effect and to characterize the source of infectious KSHV virions in saliva.

## Data Availability

All raw data used for this manuscript is available upon request.
